# Left hepatic lobe herniation through an incisional anterior abdominal wall hernia and right adrenal myelolipoma: a case report and review of the literature

**DOI:** 10.1186/1752-1947-6-4

**Published:** 2012-01-10

**Authors:** Carlos M Nuño-Guzmán, José Arróniz-Jáuregui, Ismael Espejo, Jesús Valle-González, Hernán Butus, Alejandro Molina-Romo, Rodrigo I Orranti-Ortega

**Affiliations:** 1 Department of General Surgery, Calle Hospital 278, Guadalajara, Jalisco, México, CP 442801; 2 Department of Pathologic Anatomy, Calle Hospital 278, Guadalajara, Jalisco, México, CP 442802

## Abstract

**Introduction:**

Herniation of the liver through an anterior abdominal wall hernia defect is rare. To the best of our knowledge, only three cases have been described in the literature.

**Case presentation:**

A 70-year-old Mexican woman presented with a one-week history of right upper quadrant abdominal pain, nausea, vomiting, and jaundice to our Department of General Surgery. Her medical history included an open cholecystectomy from 20 years earlier and excessive weight. She presented with jaundice, abdominal distension with a midline surgical scar, right upper quadrant tenderness, and a large midline abdominal wall defect with dullness upon percussion and protrusion of a large, tender, and firm mass. The results of laboratory tests were suggestive of cholestasis. Ultrasound revealed choledocholithiasis. A computed tomography scan showed a protrusion of the left hepatic lobe through the anterior abdominal wall defect and a well-defined, soft tissue density lesion in the right adrenal topography. An endoscopic common bile duct stone extraction was unsuccessful. During surgery, the right adrenal tumor was resected first. The hernia was approached through a median supraumbilical incision; the totality of the left lobe was protruding through the abdominal wall defect, and once the lobe was reduced to its normal position, a common bile duct surgical exploration with multiple stone extraction was performed. Finally, the abdominal wall was reconstructed. Histopathology revealed an adrenal myelolipoma. Six months after the operation, our patient remains in good health.

**Conclusions:**

The case of liver herniation through an incisional anterior abdominal wall hernia in this report represents, to the best of our knowledge, the fourth such case reported in the literature. The rarity of this medical entity makes it almost impossible to specifically describe predisposing risk factors for liver herniation. Obesity, the right adrenal myelolipoma mass effect, and the previous abdominal surgery are likely to have contributed to incisional hernia formation.

## Introduction

Herniation of the liver is uncommon. This entity has been described in patients with omphalocele, congenital diaphragmatic hernia, and blunt trauma diaphragmatic rupture [1-3]. To the best of our knowledge, only three cases of liver herniation through an anterior abdominal wall hernia have been described [4-6]. The case of an obese woman who had an open cholecystectomy 20 years earlier is reported here. A midline abdominal wall hernia with a protrusion of the left hepatic lobe through the hernia defect and a large mass in the right adrenal gland were found.

## Case presentation

A 70-year-old Mexican woman presented with a one-week history of right upper quadrant abdominal pain, nausea, vomiting, and jaundice to our Department of General Surgery. Her medical history included an open cholecystectomy, which was performed 20 years earlier in another hospital to treat acute cholecystitis, and excessive weight during the past 45 years.

A physical examination revealed jaundice, abdominal distension with a midline surgical scar, right upper quadrant tenderness, and a large abdominal wall defect suggestive of a post-incisional hernia with protrusion of a large, tender, and firm mass. Percussion elicited dullness over the entire hernia content. No rebound tenderness, shifting dullness, or fluid wave was observed.

Laboratory tests revealed a total bilirubin value of 3.2 mg/dL (reference value of 0.2 to 1.3), direct bilirubin of 2.2 mg/dL (reference value of 0.1 to 0.5), alkaline phosphatase of 415 U/L (reference value of 40 to 150), gamma-glutamyl transferase of 241 U/L (reference value of 9 to 64), alanine aminotransferase of 139 U/L (reference value of 0 to 50), and aspartate aminotransferase of 142 U/L (reference value of 0 to 40).

**Figure 1:**
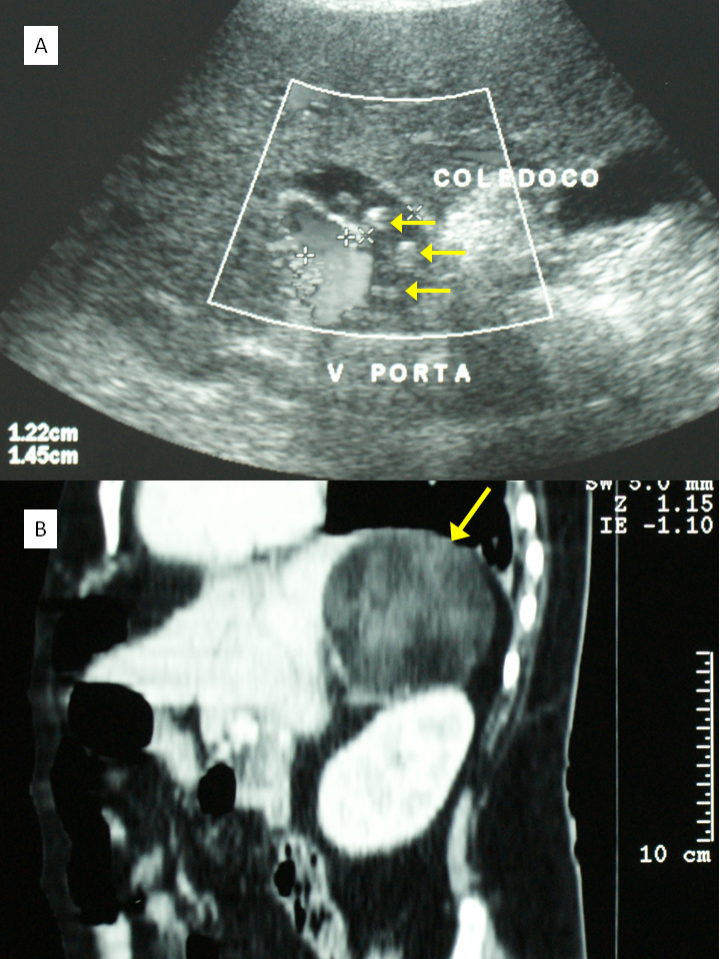
**(A) Ultrasound aspect of dilated common bile duct with images suggestive of choledocholithiasis (arrows)**. (B) Computed tomography saggital view showing the mass in the right adrenal topography (arrow).

**Figure 2:**
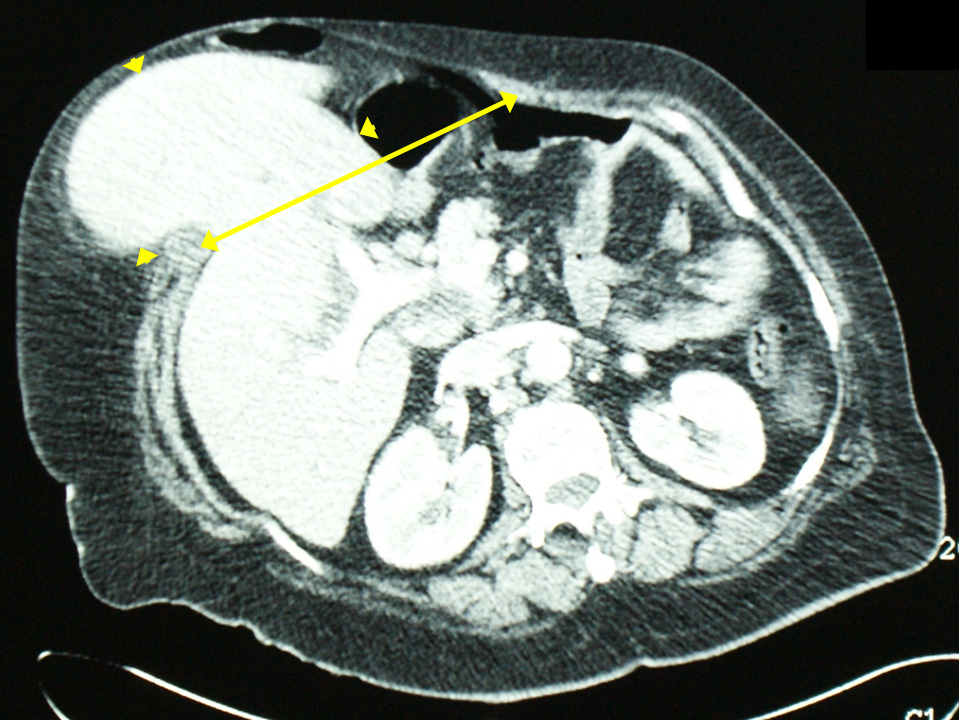
**Protrusion of the left hepatic lobe (arrow heads) through the abdominal wall defect (arrow) on axial view of computed tomography**.

During transabdominal ultrasound (US), intrahepatic and extrahepatic bile duct dilatation and stones in the common bile duct were observed (Figure 1A). A computed tomography (CT) scan revealed a midline abdominal wall hernia defect with protrusion of the left hepatic lobe (Figure 2). A large, well-defined, soft tissue density lesion with intrinsic fat density areas in the right adrenal topography was identified (Figure 1B). Endoscopic stone extraction through an endoscopic retrograde cholangiopancreatography (ERCP) was not possible (because of duodenal compression by the herniated left hepatic lobe), and the obesity of our patient made it difficult to keep her in a prone position. As a result, Vater ampulla cannulation was unsafe and very difficult.

**Figure 3:**
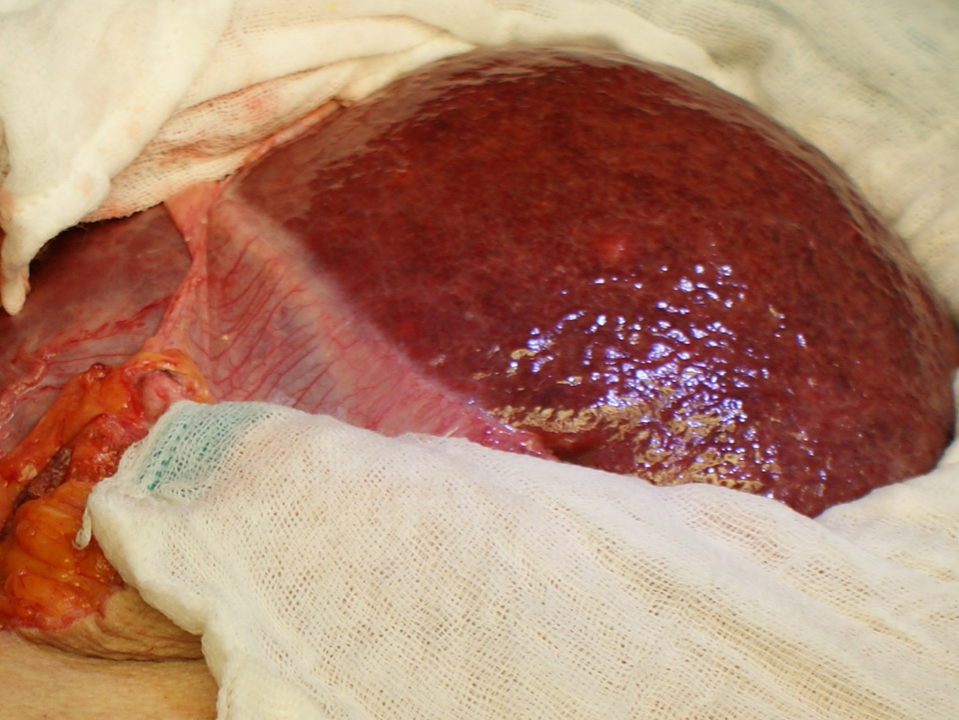
**Surgical aspect once the left hepatic lobe was dissected and the hernia content was reduced**.

Our patient was scheduled for surgery. The right adrenal tumor was approached and resected through a right subcostal incision. The hernia was approached through a median supraumbilical incision. A left lobe protrusion through the abdominal wall defect was found, and a reduction of the lobe to its normal position was performed (Figure 3). The common bile duct appeared dilated, and after duodenum mobilization through a Kocher maneuver, a longitudinal choledochotomy was performed between two 5-0 stay sutures in the anterior surface of the common bile duct. Multiple stones were extracted, and after Vater ampulla patency was demonstrated, a T-tube was placed, and the choledochotomy was closed with interrupted 5-0 absorbable sutures. Cholangiography was performed through the T-tube.

**Figure 4:**
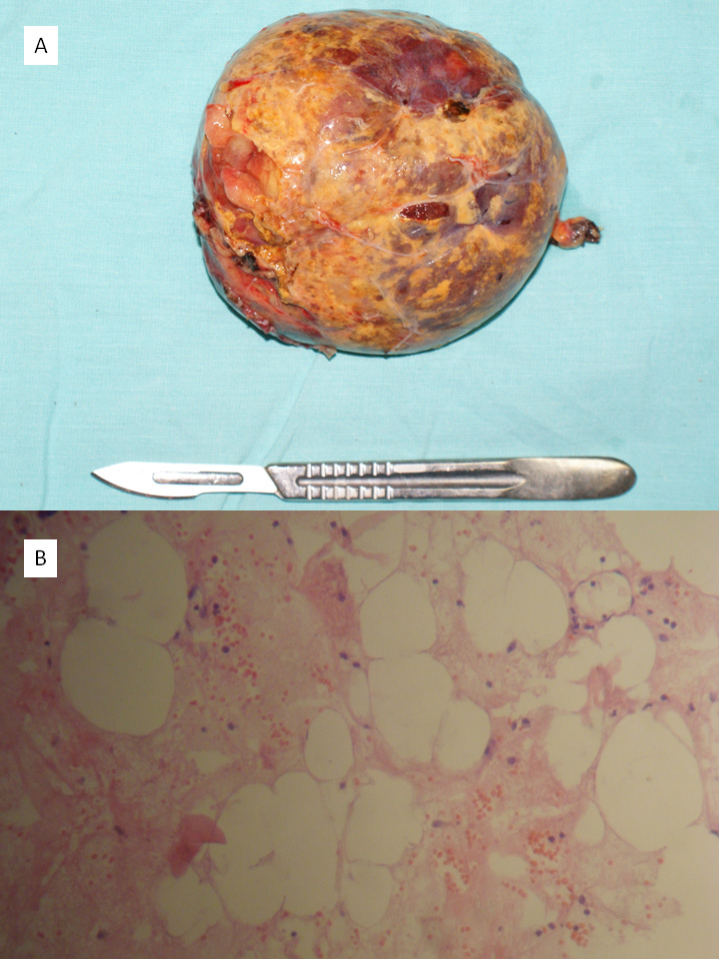
**(A) Macroscopic aspect of the right adrenal mass**. (B) Histology demonstrated a solid tumor formed by well-differentiated adipose cells, fibrous stroma in regular septa, and lymphoid cells, indicative of an adrenal myelolipoma (hematoxylin and eosin, ×10).

Macroscopically, a 12cm, yellowish, soft tumor was found (Figure 4A). Histopathology revealed a solid tumor formed by completely differentiated adult-type adipose cells, fibrous stroma in regular septa with scarce venous capillaries, and scattered or grouped lymphoid cells, indicative of an adrenal myelolipoma (Figure 4B). Our patient required supplementary oxygen during the first post-operative day and was discharged seven days after the surgery. Six months after the operation, she remains in good health.

## Discussion

Herniation of the liver is an uncommon situation. An entity known as exclusive hepatocele, in which the liver is part of the omphalocele content, has been described in the neonatal period [1]. Compromise of the right hepatic lobe in a right-sided Bochdalek hernia has also been reported. Diagnosis in adult patients is extremely rare [2]. Liver herniation through a blunt trauma diaphragmatic rupture is rare because of the energy-absorbing effect of the liver below the right hemidiaphragm [3]. Liver herniation through an anterior abdominal wall hernia is much less common [4-6].

Sheer and Runyon [4] reported the case of a 45-year-old woman who had a laparotomy for trauma 33 years earlier and an orthotopic liver transplantation two years earlier. She presented with confusion and progressive upper abdominal pain and swelling for the previous three months. A CT scan showed hepatomegaly and a fatty-infiltrated liver protruding through an incisional wall defect. She was admitted to the intensive care unit and died of *Pseudomonas *sepsis [4].

Abci and colleagues [5] reported the case of a 73-year-old woman who had a cholecystectomy through a right subcostal incision six years earlier and a laparotomy for intestinal obstruction four years earlier. The patient had a six-month evolution of right upper quadrant abdominal pain, nausea, and dyspnea. A physical examination revealed a right 3 × 3 cm zone of induration at the subcostal surgical scar but no rebound tenderness. On a CT scan, an incarcerated incisional hernia associated with the medial segment of the left hepatic lobe was identified. Owing to cardiac and pulmonary disease in the absence of peritonitis, the patient was managed non-surgically [5].

Shanbhogue and Fasih [6] reported the case of a 48-year-old woman with a three-week history of discomfort and swelling in the epigastrium. Two years earlier, the patient had coronary artery bypass surgery that was further complicated by post-surgical sternal dehiscence. She had a lump on the epigastrium with minimal tenderness. A CT scan showed herniation of a left hepatic lobe segment through a midline defect in the anterior abdominal wall. Since her symptoms were minor, she was not operated on [6].

Rodríguez-Hermosa and colleagues [7] reported a case not of a hernia but of a right liver lobe evisceration in a superobese patient (body mass index of 57 kg/m^2^) after an emergency laparoscopic surgery for acute calculous cholecystitis was converted to open surgery.

Herniation of the liver through other types of incisional hernias has been described. Salemis and colleagues [8] reported the case of a 58-year-old woman who had a right nephrectomy via a retroperitoneal approach through a flank incision, after which she developed a right incisional lumbar hernia. Once the hernia was repaired, she presented with a recurrent hernia, which became incarcerated. On magnetic resonance (MR), a right liver lobe segment was observed [8]. Losanoff and colleagues [9] reported the case of a recurrent intercostal herniation of the liver. Adeonigbagbe and colleagues [10] reported a case in which the herniation of a liver segment through the rectus muscle presented as persistent abdominal pain.

Adrenal myelolipomas are uncommon, benign, unilateral, and non-functioning tumors composed of mature adipose tissue admixed with hematopoietic cells [11]. As referred by Plaut, [12] Gierke was the first to recognize circumscribed bone marrow-like structures in the adrenal gland, whereas Oberling named them myelolipomatous formations. Myelolipomas account for approximately 3% to 5% of all primary adrenal tumors. The incidence is estimated at 0.08% to 0.25% [13]. Mean ages at diagnosis of 50 and 62 years have been reported in different series, and no gender predilection has been noted. These lesions are usually asymptomatic and incidentally found through CT scanning for other reasons or at autopsy. Myelolipomas are often less than 4 cm in diameter when discovered but can attain very large sizes. Back, flank, or abdominal vague pain with or without a palpable mass and hematuria has been observed. Macroscopically, myelolipoma is a non-encapsulated, bright yellow, well-circumscribed lesion with foci of tan-brown discoloration. Microscopically, this lesion is composed of mature adipose tissue with scattered islands of hematopoietic cells. Areas of necrosis, hemorrhage, cyst formation, and calcification or ossification may also be evident, particularly in larger tumors [11,13]. After excision, they generally do not recur. These tumors are generally hormonally inactive, although hormone overproduction has been reported.

Diagnosis of adrenal myelolipomas is made by US, CT, and MR in more than 90% of cases. CT is the most sensitive diagnostic imaging modality [13]. In US, a myelolipoma typically appears as a hyperechoic mass because of its fatty and myeloid tissue constituents whereas areas of pure fat may be hypoechoic. The tumor margins may be difficult to define [14]. In CT, the lesion appears as a well-defined heterogeneous mass with low-density mature fat and a more dense myeloid tissue surrounded by a fine capsule. This tumor typically has an attenuation that ranges from -30 to -115 Hounsfield units and that is significantly lower than that of cortical adenomas. Enhancement in the soft tissue component of the tumor is observed after contrast material intravenous administration [13-15]. In MR, this tumor shows a high signal intensity on T1-weighted and T2-weighted images equal to that on images of subcutaneous and retroperitoneal fat. Similar to the spleen, admixed marrow-like elements have medium signal intensity [13,14]. Calcification can be seen in up to 27% of adrenal myelolipomas. The tumors are generally large when they are discovered, in part because they are asymptomatic until compression of adjacent structures. To make a diagnosis that cannot be confidently made by means of non-invasive imaging or to rule out malignancy, fine needle aspiration biopsy should be considered [13].

Histologic diagnosis of myelolipoma is based on the presence of mature adipocytes and hematopoietic elements. Treatment modalities include watchful waiting and resection. Small asymptomatic tumors can be monitored expectantly since they pose little risk of spontaneous rupture or bleeding. Often, although these tumors are benign, their size and propensity to grow warrant surgical removal. Symptomatic tumors and those rare hormonally active tumors should be excised. Smaller tumors are amenable to laparoscopic resection [13].

The clinical presentation of liver herniation through an anterior abdominal wall hernia tends to be non-acute with no peritonitis and probably is associated with the large hernia defect and the absence of strangulation. The decision to perform an abdominal wall reconstruction should be made on an individual basis. In the case reported here, the one-week clinical presentation of abdominal pain and jaundice, the previous cholecystectomy, and the liver biochemical markers made a common bile duct obstruction highly probable, and this was confirmed by US. The difficulty of performing a successful stone extraction by ERCP because of the patient's obesity and duodenal compression by a displaced liver and the right adrenal tumor made the surgical decision mandatory. In our case, obesity and the mass effect caused by the adrenal myelolipoma are likely to have contributed to an increased intra-abdominal pressure, which was exerted over a previously operated abdominal wall, with subsequent incisional hernia formation. Although a common background of major surgery is present in the three cases of liver herniation through an anterior incisional defect as well as in our case, the rarity of this medical entity makes it almost impossible to specifically describe predisposing risk factors for liver herniation.

## Conclusions

The case of liver herniation through an incisional anterior abdominal wall hernia in this report represents, to the best of our knowledge, the fourth such case reported in the literature. Obesity, the right adrenal myelolipoma mass effect, and the previous surgical event are likely to have contributed to the incisional hernia formation. The description of the diagnostic approach and the consideration of comorbidities in this and previous case reports are important for such rare cases.

## Abbreviations

CT: computed tomography; ERCP: endoscopic retrograde cholangiopancreatography; MR: magnetic resonance; US: ultrasound.

## Consent

Written informed consent was obtained from the patient for publication of this case report and any accompanying images. A copy of the written consent is available for review by the Editor-in-Chief of this journal.

## Competing interests

The authors declare that they have no competing interests.

## Authors' contributions

CMNG and JAJ were involved in the medical care of the patient and helped to write the manuscript. JVG, HB, AMR, and RIOO were involved in the medical care of the patient and contributed to the literature review. IE performed the histological examination of the myelolipoma and contributed to the literature review. All authors read and approved the final manuscript.
